# New biomarkers of inflammation associated with haemodialysis

**DOI:** 10.1093/ckj/sfaf223

**Published:** 2025-07-10

**Authors:** Fátima Guerrero, Andrés Carmona, Maria Jose Jiménez, Francisco Ariza, Teresa Obrero, Isabel Berdud, Carolina Carrillo-Carrión, Mariano Rodríguez, Sagrario Soriano, Juan R Muñoz-Castañeda, Alejandro Martín-Malo

**Affiliations:** Maimonides Biomedical Research Institute of Cordoba (IMIBIC), University of Córdoba, Reina Sofía University Hospital, Córdoba, Spain; Maimonides Biomedical Research Institute of Cordoba (IMIBIC), University of Córdoba, Reina Sofía University Hospital, Córdoba, Spain; Maimonides Biomedical Research Institute of Cordoba (IMIBIC), University of Córdoba, Reina Sofía University Hospital, Córdoba, Spain; Maimonides Biomedical Research Institute of Cordoba (IMIBIC), University of Córdoba, Reina Sofía University Hospital, Córdoba, Spain; Dialysis Satellite Unit, Fresenius Medical Care Services Andalucía, Córdoba, Spain; Maimonides Biomedical Research Institute of Cordoba (IMIBIC), University of Córdoba, Reina Sofía University Hospital, Córdoba, Spain; Dialysis Satellite Unit, Fresenius Medical Care Services Andalucía, Córdoba, Spain; Institute for Chemical Research (IIQ), CSIC-University of Seville, Seville, Spain; Maimonides Biomedical Research Institute of Cordoba (IMIBIC), University of Córdoba, Reina Sofía University Hospital, Córdoba, Spain; Maimonides Biomedical Research Institute of Cordoba (IMIBIC), University of Córdoba, Reina Sofía University Hospital, Córdoba, Spain; Nephrology Service, Reina Sofia University Hospital, Córdoba, Spain; Maimonides Biomedical Research Institute of Cordoba (IMIBIC), University of Córdoba, Reina Sofía University Hospital, Córdoba, Spain; Nephrology Service, Reina Sofia University Hospital, Córdoba, Spain; Maimonides Biomedical Research Institute of Cordoba (IMIBIC), University of Córdoba, Reina Sofía University Hospital, Córdoba, Spain

**Keywords:** cardiovascular disease, haemodialysis, inflammation, monocytes, uraemic toxins

## Abstract

**Background:**

The first year of haemodialysis (HD) carries the highest risk of mortality, which to a large extent is attributed to the aggravation of inflammation. However, traditional markers such as C-reactive protein and interleukin-6 show only minor changes during the first year, suggesting that there are other factors involved. The present study evaluates the effect of HD on microinflammation and oxidative stress of uremic patients.

**Methods:**

We conducted a prospective observational longitudinal study including 30 incident HD patients. Blood samples were collected at baseline and 6 and 12 months. Pro-inflammatory monocytes were quantified using flow cytometry. Proteomic analysis (Olink) was performed on serum. Concentrations of indoxyl sulphate (IS), growth differentiation factor 15 (GDF-15), oxidative status and circulating microRNA (miRNA) expression were also determined.

**Results:**

A new population of activated monocytes was identified that progressively increased at 1 year of HD. In addition, an increase in the serum concentration of up to 29 inflammation-related proteins was detected, including interleukins, chemokines, tumour necrosis factor family molecules, cell activation molecules and apoptosis-related proteins. Conversely, leukaemia inhibitory factor receptor was downregulated. The concentration of IS was positively correlated with GDF-15 levels. Furthermore, patients exhibited decreased expression of miRNA-126-3p, -130a-3p, -146a-5p, 223-3p, -let7a-5p and -let7b-5p.

**Conclusion:**

This study highlights the impact of HD on inflammation and oxidative stress, manifested by an increase in activated monocytes and inflammatory markers. The observed subclinical inflammation associated to HD treatment may help in understanding the mechanisms of cardiovascular damage in patients on HD.

KEY LEARNING POINTS **What was known:**

- Chronic kidney disease (CKD) is currently a major global public health problem worldwide.
- Microinflammation, a hallmark of CKD, plays a critical role in the development and progression of cardiovascular disease.
- Traditional inflammation markers such as C-reactive protein and interleukin-6 have been extensively evaluated in HD patients, but they alone cannot fully explain the high cardiovascular mortality observed in patients during the first year of haemodialysis (HD).
- It is important to identify new biomarkers to better understand the complex inflammatory process in HD patients.

**This study adds:**

- A monocyte population characterized by high forward scatter in flow cytometry and elevated expression of CD16 has been identified in HD patients and they increase progressively during the first year of HD.
- Inflammatory status and oxidative stress results changed significantly after the first year of HD.
- Circulating levels of indoxyl sulphate were positively correlated with the concentration of growth differentiation factor 15, a systemic inflammation marker.

**Potential impact:**

- The findings of the present study add valuable insights on HD-related inflammation and cardiovascular processes, a necessary step to improve the care of our HD patients.

## INTRODUCTION

Chronic kidney disease (CKD) is currently a major health problem that affects ≈13% of the adult population [1]. Haemodialysis (HD), the most common form of kidney replacement therapy, is a life-saving treatment for patients with end-stage renal disease (ESRD). While HD can prolong the life of patients with CKD, it is also potentially associated with several complications, including an increased risk of cardiovascular disease (CVD). In fact, CVD is recognized as a major cause of mortality in HD patients [2]. The underlying mechanisms are directly related to the high incidence of risk factors in this population, including persistent inflammation, endothelial dysfunction, oxidative stress and vascular calcification [3]. In addition, oxidative stress and inflammation are common in CKD patients, particularly in those undergoing HD, and there are conditions associated with the development of CVD [4].

The first year after initiation of HD represents the period of highest mortality risk, mainly due to cardiovascular causes, which is explained by worsening of the overall inflammatory state [5]. Among the pro-inflammatory factors, cytokines are crucial in the regulation of immune responses and in the progression of inflammatory diseases. Among the traditional markers of inflammation, C-reactive protein (CRP) and interleukin-6 (IL-6) have been extensively evaluated in HD patients. Previous studies evaluating inflammatory and oxidative stress–related parameters in incident HD patients have not observed significant changes in traditional inflammation-related markers [6–8]. Therefore, the question arises as to whether yet unidentified biomarkers might play a significant role in modulating the inflammatory state in HD patients, potentially contributing to the alteration of factors that promote the development of CVD, such as endothelial dysfunction, senescence or oxidative stress.

Monocytes are recognized as key contributors to chronic systemic inflammation, endothelial injury and accelerated atherosclerosis in patients with CKD, as previously reported by our group and others [9, 10]. Recent studies revealed the presence a new population of atypical monocytes characterized by high forward scatter (FSC; by flow cytometry analysis), which had been overlooked so far and may provide some clues about the inflammatory process [11]. These monocytes exhibit distinct phenotypic and functional properties, including modified cytokine profiles, increased expression of adhesion molecules and enhanced migratory capacity, which could also notably contribute to inflammation-induced CVD in HD patients. Thus we must improve our understanding of the complex inflammatory process in such patients. In this context we conducted a prospective observational study in incident HD patients to assess the effect of long-term HD on the inflammatory state. To achieve this, we performed a novel proteomic assay focused on inflammatory proteins and measured additional soluble markers of inflammation and oxidative stress, including growth differentiation factor 15 (GDF-15), circulating monocyte phenotypes (morphology and activation status), oxidative stress and expression of specific microRNAs (miRNAs). Our aim was to identify new key biomarkers and explore potential relationships between new and traditional markers that could enhance our ability to predict the prognosis of ESRD patients undergoing routine HD.

## MATERIALS AND METHODS

All procedures were performed in accordance with the ethical standards of the institutional research committee and complied with the standards established by the latest revision of the Declaration of Helsinki and the Council of Europe Convention (1996) concerning human rights and biomedicine. The study protocol was approved by the local institutional ethics committee (Comité de Ética de la Investigación de Córdoba; ref. 3756, protocol code PI17/01785). All patients provided written informed consent to participate in the study.

### Patients

The study included 30 consecutive ESRD patients who started HD treatment at the Nephrology Service of Reina Sofía University Hospital and the dialysis satellite centre in Córdoba and completed the first 12 months of HD without cardiovascular events. HD was performed using a high-permeability membrane. The dialyzers used in this study were the Phylther HF22SD (Bellco, Modena, Italy; *n* = 18) and Fx80 (Fresenius Medical Care, Hesse, Germany; *n* = 12). Patients were 18–80 years old, with a functioning vascular access (blood flow >300 mL/min), receiving three dialysis session per week for a duration of 4–4.5 h, providing an adequate dose of dialysis (eKt/v >1.5) and with no evidence of cardiovascular events during the 2 months prior to inclusion. Patients with clinical signs of active neoplasm, diagnosis of autoimmune disease, positive viral markers (hepatitis B surface antigen, anti-hepatitis C virus and human immunodeficiency virus) or inflammatory processes, defined as the presence of clinical evidence of active inflammation, such as a reported history of fever, pain or other symptoms suggestive of an ongoing inflammatory process, were excluded from this study.

### Blood sampling

Blood samples were collected at baseline (just before starting the first HD session) and at 6 and 12 months after starting their regular HD. Ten age- and gender-matched healthy subjects were used as controls. A sample of peripheral blood was drawn from the blood line before the midweek HD session from patients and from a peripheral vein in healthy subjects; blood samples were collected into tubes containing ethylenediaminetetraacetic acid or clot activator (SST). Blood was collected carefully to minimize the time of processing and prevent platelet activation. The samples were maintained under a controlled temperature (4–8°C) and processed within a short period of time, usually <2 h after blood sample collection. The blood was centrifuged at 1500 *g* for 10 min at room temperature to obtain platelet-poor plasma and serum that were subsequently aliquoted into polypropylene tubes in 0.5-mL volumes and stored at −80°C until analysis.

### Monocyte subpopulations

To assess expression of surface markers in monocyte subpopulations by flow cytometry, fluorescein isothiocyanate–conjugated anti-CD14 (MHCD1401; Invitrogen, Waltham, MA, USA) and allophycocyanin-conjugated anti-CD16 (MHCD1605; Invitrogen) were used.

Immunolabeling and flow cytometry were performed in whole blood to avoid centrifugation and washing steps, which may cause artifactual platelet activation. About 200 μl of whole blood were added to polystyrene round-bottom tubes and incubated with two different antibody combinations for 20 min at room temperature and in dark conditions. Thereafter, red blood cells were lysed by the addition of 500 μl of FACS-Lysing solution (349202; Becton Dickinson, San Jose, CA, USA) for 15 min at room temperature in the dark. Finally, the samples were fixed with 500 μl of CellFix (340181; Becton Dickinson). Quickly, stained cells were acquired on an LSRFortessa flow cytometer (Becton Dickinson). An acquisition threshold was set in the FSC parameter and unwanted elements like platelets, dead cells and debris were not recorded. Analysis was performed using FlowJo software (version 10.9.0; Becton Dickinson).

**Figure 1:**
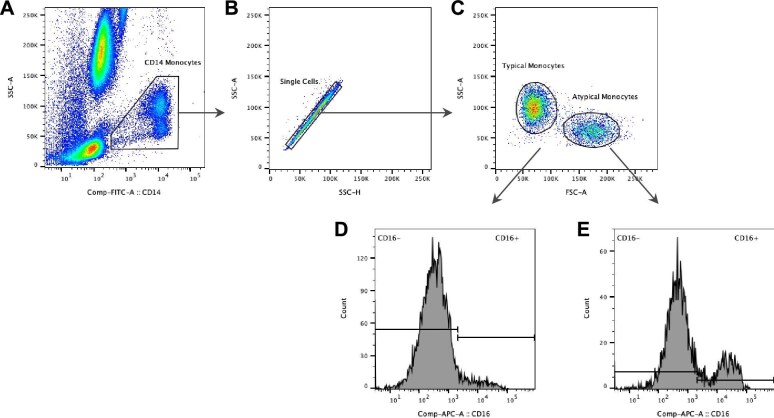
Gating strategy for monocyte subset determination. **(A)** CD14^high^/SSC-A (side scatter area) was used to identify monocyte cells among other leucocytes. **(B)** Doublets were removed by gating on SSC-A versus SSC-H (side scatter height). **(C)** Forward and side scatter area (FSC-A/SSC-A) identified typical and atypical monocytes. **(D, E)** Expression of surface marker CD16 was analysed in gated cells.

The gating strategy used is summarized in Fig. 1. Briefly, the monocytes were identified based on their side scatter (SSC) characteristic versus the surface marker CD14. On the monocyte gate, a plot of the area versus the measurement of height was used to remove clumps of cells, which show up with increased area relative to the height. The singlet gate was further visualized in an FSC and SSC plot. Next, pro-inflammatory monocytes were identified based on CD16 expression (CD16^+^). The expression of surface markers was evaluated by calculating the median fluorescence intensity (MFI) of the gated cell population.

### Analytical methods

A panel of 92 circulating inflammatory proteins were determined using a multiplex proximity extension assay from Olink (COBIOMIC Bioscience, Cordoba, Spain). Moreover, commercial enzyme-linked immunosorbent assay kits were used to quantify the levels of GDF-15 (DGD150; R&D Systems, Minneapolis, MN, USA), IS (LGM-18002; Leadgene Biomedical, Taiwan), total antioxidant capacity (TAC; STA-360; Cell Biolabs, San Diego, CA, USA) and malondialdehyde (MDA; KB03016; Bioquochem, Asturias, Spain) following the protocol provided by the manufacturer.

### Expression of miRNA

Total RNA, including the miRNA fraction, was extracted from serum using a commercial, column-based system (miRNeasy Serum/Plasma Advanced Kit; 217204; Qiagen, Hilden, Germany) according to the manufacturer's protocol with minor modifications. Expression levels of circulating miRNA was quantified by reverse transcription (miRCURY LNA RT Kit; 339340; Qiagen) and real-time quantitative polymerase chain reaction (miRCURY LNA SYBR Green PCR Kit; 339346; Qiagen). The cel-miR-39-3p RNA Spike-in was included in the reaction of control for complementary DNA synthesis. The amplification was performed in a LightCycler 96 Real-Time PCR System (Roche, Basel, Switzerland). Cycling conditions were as follows: 95°C for 120 sec, followed by 45 cycles at 95°C for 10 sec and 56°C for 60 sec. Melt curve analysis was performed between 60°C and 95°C at a ramp rate of 0.20°C/sec. The amplification curves were analysed using LightCycler 96 software (version 1.1; Roche). The expression of miRNA was calculated by subtracting the control values (Ct) of the exogenous control from the Ct values of the miRNA of interest (Ct miRNA − Ct cel-miR-39-3p).

The identification assays used were hsa-miR-126-3p (YP00204227), hsa-miR-130a-3p (YP00204658), hsa-miR-146a-5p (YP00204688), hsa-miR-223-3p (YP00205986), hsa-miR-let7a-5p (YP00205727), hsa-miR-let7b-5p (YP00204750) and cel-miR-39-3p (YP00203952) (Qiagen).

### Statistical analysis

The data distribution was analysed with the Shapiro–Wilk test. Comparisons of values obtained in the same patients at three time points were performed using a one-way analysis of variance followed by a Bonferroni post hoc test if the data were normally distributed, otherwise the Wilcoxon signed-rank test was used. Differences between means for unpaired data were analysed using either an unpaired *t*-test or a Mann–Whitney U test, as appropriate. Changes over time in monocyte variables were assessed using linear mixed models. Spearman’s correlation test was used to assess the associations between variables. We utilized GraphPad Prism version 6.0 (GraphPad Software, Boston, MA, USA) and SPSS version 20.0 (IBM, Armonk, NY, USA) for statistical analysis, visualization and correlation studies. The results are presented as mean ± standard error of the mean (SEM). *P*-values <.05 were considered statistically significant. Volcano plots were used to visualize the mean differences and *P*-values, with a significance threshold set at 0.05, identifying proteins with both substantial changes and statistical significance. Heat maps represented protein expression levels across different time points, providing a comprehensive view of temporal dynamics. For pathway enrichment analysis, we utilized the STRING database and Appyters platform, identifying potentially relevant biological pathways.

## RESULTS

### Baseline clinical characteristics

The study included a cohort of 30 incident patients, 23 male (77%) and 7 female (23%), with an average age of 65.4 ± 16.5 years. The clinical characteristics are presented in detail in Table 1.

**Table 1:** Baseline clinical characteristics of the 30 ESRD patients included in the study.

Clinical characteristics	Values, mean ± SEM
Haemoglobin (g/dl)	10.4 ± 0.2
Transferrin saturation (%)	25.2 ± 1.4
Iron (μg/dl)	57.8 ± 3.4
Urea (mg/dl)	198.8 ± 12.7
Creatinine (mg/dl)	6.2 ± 0.3
Calcium (mg/dl)	8.2 ± 0.2
Phosphorus (mg/dl)	5.3 ± 0.2
C-reactive protein (mg/dl)	13.2 ± 3.8
Beta-2 microglobulin (mg/l)	16.7 ± 0.8
Albumin (g/dl)	3.8 ± 0.1
Magnesium (mg/dl)	2.1 ± 0.1

### Subpopulations of circulating monocytes

**Figure 2:**
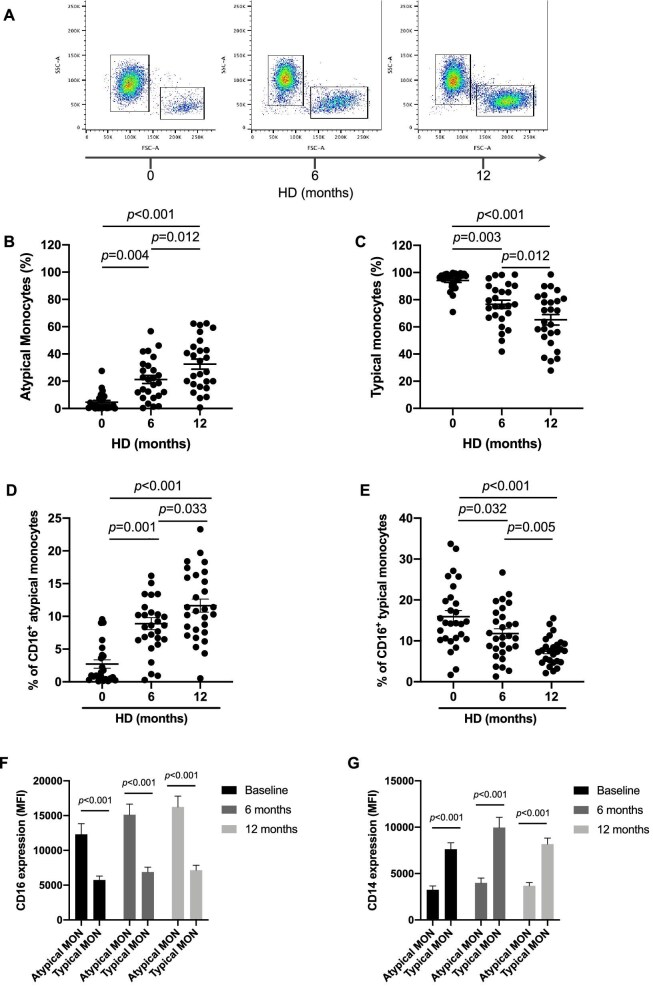
Monocyte subsets in blood from incident HD patients for 12 months of follow-up. **(A)** Representative FACS plots depicting monocyte subsets. The *x*-axis represents the forward scatter area (FSC-A), with higher signal indicating an increase in cell size. **(B)** Percentage of atypical monocytes and **(C)** typical monocytes at baseline, 6 months and 12 months following the initiation of HD treatment. Percentage of pro-inflammatory CD16^+^ cells within **(D)** atypical monocytes and **(E)** typical monocytes. **(F)** CD16 and **(G)** CD14 expression levels on typical and atypical monocytes quantified by changes in the MFI. Data are presented as median ± SEM. *P*-values <.05 were considered on the borderline of statistical significance.

The percentage of peripheral blood monocytes did not show significant differences in the three time periods studied (6.3 ± 0.3, 6.8 ± 0.3 and 6.7 ± 0.3 at 0, 6 and 12 months respectively). However, if monocytes are analysed according to their size (Fig. 2A), the percentage of large monocytes (high FSC values; atypical monocytes) progressively increases during the first 12-month period of HD (4.7 ± 1.3, 21.3 ± 2.9 and 32.5 ± 3.7 at 0, 6 and 12 months, respectively; *P* < .05) (Fig. 2B). In contrast, the percentage of normal-size monocytes (low FSC values; typical monocytes) decreased significantly throughout the initial 12-month period of HD (94.1 ± 1.3, 76.6 ± 3.5 and 65.2 ± 3.8, respectively; *P* < .05) (Fig. 2C).

While the percentage of CD16^+^ atypical monocytes increased significantly during the initial period of HD (2.7 ± 0.6, 8.9 ± 0.9 and 11.6 ± 1.0 at baseline, 6 and 12 months, respectively; *P* < .05) (Fig. 2D), the percentage of CD16^+^ typical monocytes decreased significantly during the initial 12-month follow-up period (15.9 ± 1.5, 11.8 ± 1.1 and 7.5 ± 0.6, respectively; *P* < .05) (Fig. 2E).

Additionally, the surface marker expression of monocytes was analysed. Atypical monocytes showed greater CD16 receptor expression than typical monocytes (Fig. 2F), and the expression of the CD14 receptor in the atypical monocytes was reduced as compared with typical monocytes (Fig. 2G). The decrease in CD14 expression in atypical monocytes suggests that this subpopulation does not reflect cell aggregates caused by cellular adhesion (Supplementary Fig. S1). Moreover, using a linear mixed model with the patient as a random factor, significant differences in the outcomes were observed (*P* < .01), suggesting that our results are not influenced by the specific characteristic of the patients.

### Inflammation-related biomarker analysis

**Figure 3:**
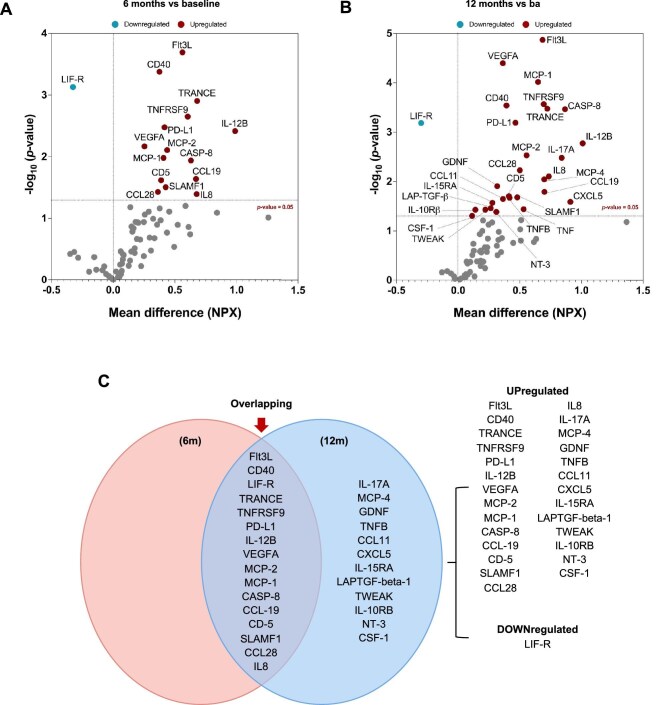
Inflammatory proteins identified in serum samples from HD patients. Volcano plot of 92 inflammation-related proteins at **(A)** 6 months and **(B)** 12 months compared with baseline in HD patients. The red points indicate significantly upregulated proteins, while blue points are significantly downregulated proteins. **(C)** Venn diagram shows differentially expressed proteins identified at 6 months, at 12 months and those common at both 6 and 12 months (overlapping region).

Proteomic profiling was assessed by a proximity extension assay (PEA; Olink). After 6 months on HD, patients presented 15 proteins that were upregulated as compared with the baseline. Only one protein, leukaemia inhibitory factor receptor (LIF-R) was downregulated at 6 months (Fig. 3A, Table 2).

**Table 2:** Differentially expressed proteins identified in serum of HD patients.

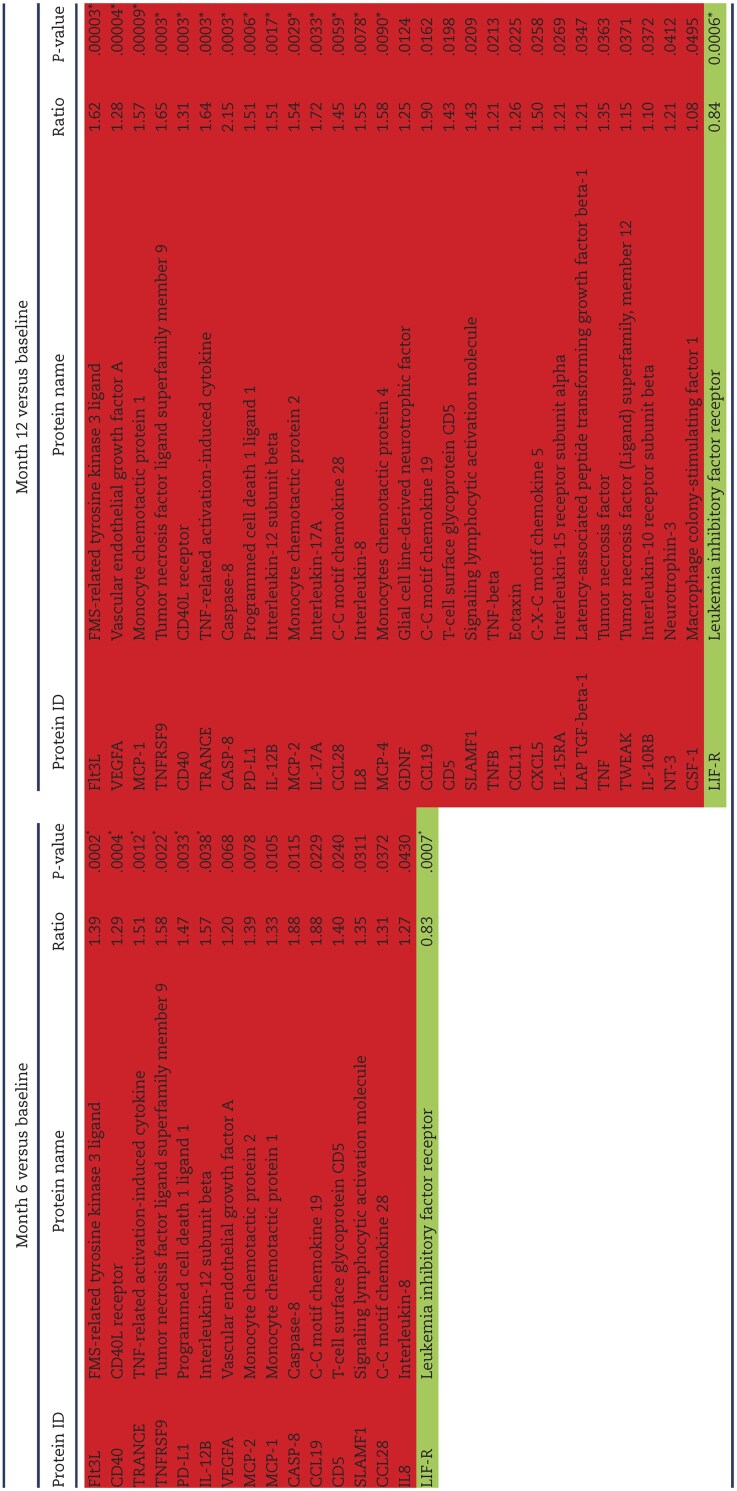

List of the upregulated (red) and downregulated (green) proteins.

^*^Proteins with a false discovery rate <0.05.

After 12 months on HD, there were 13 additional proteins that were upregulated, for a total of 28 upregulated proteins. The protein LIF-R remained to be downregulated after 12 months (Fig. 3B, Table 2). The levels of most of these proteins increased from 6 to 12 months, although differences were not significant (Fig. 3C). Interestingly, several differentially expressed proteins in the serum of HD patients at 12 months of follow-up were significantly associated with levels of CD16^+^ atypical monocytes (Table 3).

**Table 3:** Correlation analysis.

	% of CD16^+^ atypical monocytes	
Protein ID	*r* ^2^	*P*-value
FLT3L	0.4996	.0002
IL-12B	0.4498	.0010
NT-3	0.4500	.0010
CASP-8	0.3809	.0064
LAP TGF-β1	0.3689	.0084
IL-10RB	0.3533	.0118
TNFB	0.3492	.0129
PD-L1	0.3245	.0215
TNFRSF9	0.3184	.0242
TNF	0.3142	.0263
CD40	0.3052	.0311
CD5	0.3043	.0317
GDNF	0.2923	.0394
TRANCE	0.2904	.0407
VEGFA	0.2813	.0478

Pearson's correlation coefficients (*r*^2^) for the relation of CD16^+^ atypical monocytes with differentially expressed proteins.

**Figure 4:**
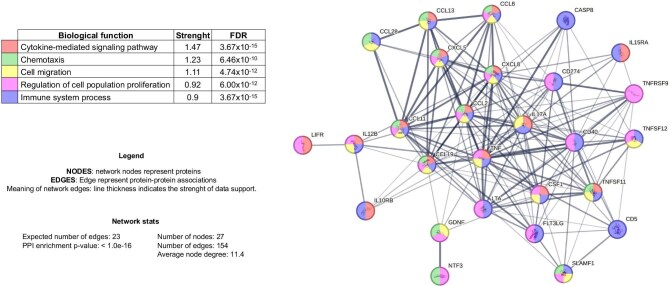
Functional classification of the altered levels of the inflammatory proteome in HD patients by STRING platform. Protein–protein interaction networks of the differentially expressed proteins in serum of HD patients.

Furthermore, enrichment analysis using the STRING platform revealed a significantly higher than expected number of interactions among these proteins as compared with a random set of proteins of similar size and degree of distribution drawn from the genome. This suggests a functional association among these proteins, which are involved in shared biological processes such as cytokine-mediated signalling, chemotaxis, cell migration, regulation of cell population proliferation and immune system processes (Fig. 4).

CRP and IL-6 levels showed no significant changes during the 1-year follow-up period. The value of CRP decreased (non-significantly) from 13.2 ± 3.8 mg/dL at baseline to 9.3 ± 1.8 mg/dL at 6 months and 9.2 ± 2.3 mg/dL at 12 months. The levels of IL-6 remained relatively stable throughout the follow-up period, with values of 4.5 ± 0.2, 4.4 ± 0.2 and 4.4 ± 0.2 normalized protein expression at baseline, 6 and 12 months, respectively.

### Changes in indoxyl sulphate (IS), GDF-15 and oxidative status

**Figure 5:**
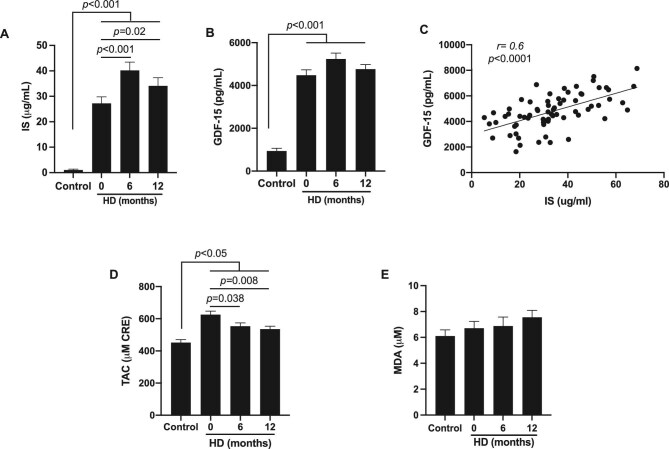
Determination of uraemic toxins, inflammation and oxidative stress markers. Serum concentrations of **(A)** IS (μg/mL) and **(B)** GDF-15 (pg/mL) in healthy subjects and HD patients. **(C)** Pearson correlation coefficient test of the relationship between serum levels of IS and levels of GDF-15. **(D)** Total antioxidant capacity levels in micromolar copper reducing equivalents (CRE) in healthy subjects and HD patients. **(E)** MDA (μM) levels in HD patients and healthy subjects. The results are presented as the mean ± SEM. *P*-values <.05 were considered on the borderline of statistical significance.

Changes in serum IS levels were assessed during the follow-up period (Fig. 5A). As expected, at the initiation of HD treatment, uraemic patients exhibited higher concentrations of IS compared with healthy subjects (27.2 ± 2.6 versus 1.1 ± 0.3 µg/mL; *P* < .001). Furthermore, our results showed a significant increase in serum levels of IS at 6 and 12 months as compared with baseline levels (40.2 ± 3.3 and 34.1 ± 3.2 versus 27.2 ± 2.6; *P* < .05).

The circulating levels of GDF-15 were significantly increased in patients with ESRD compared with healthy controls (4479.1 ± 252.8 versus 939.2 ± 129.9 µM; *P* < .001). However, no significant differences were observed during the 12-month follow-up (Fig. 5B). It is interesting to mention that circulating levels of GDF-15 showed a positive correlation with serum levels of IS (*r* = 0.6, *P* < .001) (Fig. 5C).

TAC was determined in ESRD patients on regular HD. As shown in Fig. 5D, uraemic patients presented significantly higher levels of TAC than healthy controls (625.8 ± 22.1 versus 450.9 ± 19.1 µM; *P* < .05). After 6 months, TAC levels were reduced significantly in patients undergoing HD (553.4 ± 20.7 versus 625.8 ± 22.1 µM; *P* < .05). After 12 months on HD, TAC levels were similar to those at 6 months and remained significantly higher than at baseline (535.5 ± 18.0 versus 625.8 ± 22.1 µM; *P* < .01). In addition, a trend was observed of an increase in lipid peroxidation in incident HD patients during 1 year of follow-up, as evidenced by progressive elevation of MDA levels (Fig. 5E).

### Expression profile of circulating miRNAs

**Figure 6:**
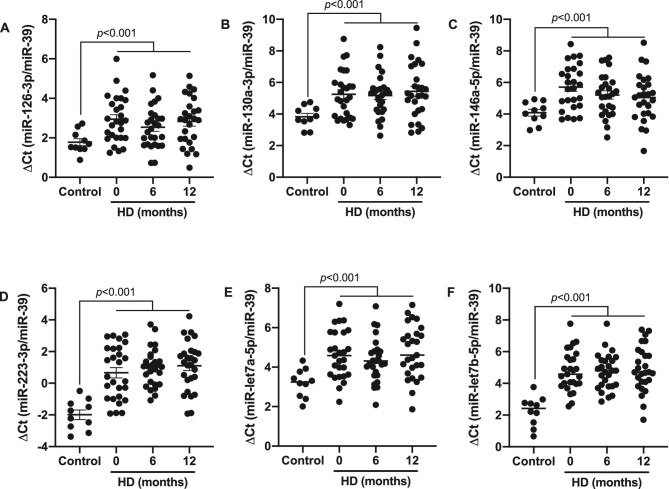
Expression levels of circulating miRNAs in HD patients. Relative miRNA expression levels of **(A)** miR-126-3p, **(B)** miR-130a-3p, **(C)** miR-146a-5p, **(D)** miR-223-3p, **(E)** miR-let7a-5p and **(F)** miR-let7b-5p in serum from patients undergoing HD. Samples were normalized to exogenous control cel-miR-39-3p. The results are presented as ΔCt values. *P*-values <.05 were considered on the borderline of statistical significance.

There were six miRNAs known to be involved in inflammation and CVD: 126-3p, 130a-3p, 146a-5p, 223-3p, let7a-5p and let7b-5p. The results are presented as ΔCt values, which exhibit an inverse relationship with the expression level, i.e. as the ΔCt value increases, the expression level decreases. Accordingly, as compared with healthy controls, in uraemic patients on maintenance HD, a significant decrease was observed in the expression levels of all analysed miRNAs (*P* < .001). However, no changes were observed from baseline to 6 and 12 months of follow-up (Fig. 6).

In our study, we analysed the impact of gender by performing a subanalysis that stratified the data by sex. The gender-stratified analysis revealed no significant differences in the evaluated parameters, except for the GDF-15 levels. Notably, it was observed that GDF-15 levels were significantly higher in males than in females (data not shown).

## DISCUSSION

This study was designed to assess the effect of the initiation of HD on circulating levels of biomarkers associated with inflammation and oxidative stress. Our results clearly indicate that as the time on HD increases, these patients present a higher percentage of pro-inflammatory atypical monocytes and increased levels of inflammation and oxidative stress markers. These findings could account to some extent for the elevated mortality rates observed in incident HD patients. Clarifying the reasons for this elevated mortality is of utmost importance, taking into account the exponential increase in patients who will need HD in the very near future. According to the World Health Organization, the prevalence of CKD is increasing globally, and it will become the fifth most common chronic non-communicable disease in 2040 [12]. CVD, which is closely linked with chronic inflammation [13], has emerged as the main cause of morbidity and mortality in this population [14]. Therefore, monitoring inflammation using specific biomarkers should aid in understanding the relationship between CKD and CVD and the HD procedure. Certainly, the identification of biomarkers should provide some clues in this complex scenario.

Monocytes are involved in the inflammatory process; the increase in circulating pro-inflammatory monocytes in CKD patients may contribute to persistent low-grade systemic inflammation by inducing endothelial damage and promoting accelerated atherosclerosis, leading to an increase in CVD risk [15]. Previous work by our group and other investigators has shown that HD treatment improves microinflammatory parameters as assessed by the percentage of CD14^+^CD16^+^ monocytes [16–18]. However, the results presented here revealed that the percentage of peripheral blood monocytes (including the total population) does not change significantly during HD treatment, at least during the 12-month period evaluated in the present study. However, different populations of monocytes may not behave uniformly. Recent investigations have focused on a new population of large monocyte cells characterized by high FSC that have been overlooked in previous studies. These larger cells have been identified as activated monocytes, showing elevated expression of the non-classical monocyte marker CD16, as well as increased expression of macrophage markers CD80 and CD206 [19]. In addition, these monocytes exhibit pronounced vacuolization and release a large amount of pro-inflammatory cytokines, such as IL-6 and tumour necrosis factor α (TNF-α), contributing to amplification of the inflammatory response that again is closely associated with the development of CVD [19, 20]. To the best of our knowledge, this novel monocyte subset has not been investigated in HD patients. Our results reveal a significant and progressive increase in the percentage of atypical monocytes as the HD treatment is sustained.

Uraemic toxins, mainly protein-bound uraemic toxins such as IS and p-cresyl sulphate, are poorly cleared by conventional dialysis [21, 22]. Numerous studies have unequivocally demonstrated that elevated levels of uraemic toxins have direct implications on systemic inflammation in uraemic patients on HD [23, 24]. Similar to previous studies [25], our results showed an increase in serum levels of IS during the 1-year follow-up period.

In this study, we evaluated whether GDF-15 levels are modified during the first year of HD therapy since GDF-15 has recently been identified as a surrogate marker of inflammation [26], oxidative stress [27] and senescence [28] and is strongly associated with cardiovascular risk and mortality in HD patients [29]. Interestingly, for the first time, a positive correlation of GDF-15 levels it was detected with the concentration of IS, a uraemic toxin that is bound to proteins.

Additionally, TAC levels were significantly increased in HD patients compared with healthy controls; this finding appears counterintuitive; however, these results are consistent with previous studies [30, 31]. Moreover, a progressive increase in MDA levels, the end-product of the lipid peroxidation process, was noted in HD patients over a 1-year follow-up. Taken together, our findings suggest the onset of increased oxidative stress in ESRD patients within the first year of kidney replacement therapy.

CRP and cytokines are classic biomarkers of chronic inflammation in patients undergoing HD. Persistent elevation of these inflammatory markers is significantly associated with adverse cardiovascular outcomes [6]. However, there are only a few studies that have assessed the longitudinal changes in these inflammatory markers during the initial period of HD treatment [6–8], and the reported results are controversial. Among these studies, Pupim *et al.* [7] did not find a significant effect of HD treatment on inflammation or oxidative stress. Specifically, they did not observe a significant increase in CRP and IL-6 during the initial 12 months of HD, suggesting that HD may not be the main cause of inflammation in these patients. More recently, Yousif *et al.* [6] observed a decrease in levels of CRP and other inflammatory parameters, such as total white blood cell count, neutrophil count, lymphocyte count and haemoglobin, whereas the serum albumin levels were increased over 36 months. In light of these non-uniform results, we decided to evaluate other new and different inflammatory parameters, among which we identified significant increases over time.

The present study found that 6 months after the initiation of regular HD there was an increase in the circulating levels of 15 proteins that are associated with inflammation, which continued to increase until the end of follow-up at month 12. Furthermore, at month 12 an increase in 13 additional proteins also associated with inflammation was observed. These findings suggest that the chronic, persistent and low-grade inflammation in incident HD patients is a dynamic process, with a gradual emergence of novel inflammatory markers likely attributed to both the HD procedure and the uraemic state. 

The upregulated proteins included pro-inflammatory cytokines such as interleukins, chemokines, TNF family molecules, cell activation molecules (e.g. SLAMF1) and apoptosis-related proteins (e.g. PD-L1, CASP-8).

Most proteins identified in this study have well-established roles in the inflammatory process associated with atherosclerosis, a main cause of CVD. Overall, monocyte chemoattractant proteins (MCPs) are key factors in the pathogenesis of endothelial inflammation-related CVD [32, 33] and contribute to the progression of atherosclerosis in HD patients [34, 35]. Members of the TNF superfamily exhibit pro-inflammatory activity through activation of the transcription factor nuclear factor κB [36]. Moreover, some members of this family, such as TWEAK, TNF-β and CD40, contribute to cell apoptosis. In fact, CD40 mediates vascular inflammation and is associated with poor cardiovascular prognosis in ESRD patients [37].

In addition, our HD patients presented an increase in serum levels of CD5, a glycoprotein that is elevated in autoimmune and inflammatory diseases. The increase in soluble CD5 is attributed to proteolytic cleavage from the membrane-bound form, a process that occurs after cell activation [38]. In our patients, a significant increase in soluble CD5 levels probably reflects chronic inflammation and immune dysregulation. For the same reason, these patients have increased production of neurotrophins, which explains our findings of increased serum levels of NT-3 and GGNF in HD patients [39, 40]. Taken together, elevated levels of these novel circulating proteins may not only indicate the presence of subclinical inflammation but may also have direct or indirect contributions in the CVD of these patients.

A state of persistent inflammation is known to modulate the expression of miRNA. In advanced CKD, there is a decrease in circulating levels miRNA mainly attributed to the accumulation of ribonucleases, leading to excessive degradation of circulating miRNA [41]. However, circulating miRNA is typically packaged in a manner that is protected from RNase activity [42]. It has been hypothesized that circulating levels of miRNA in HD patients might be associated with several factors inherent to CKD, such as uraemia, oxidative stress and inflammation, which may induce epigenetic modifications that modulate the transcription of miRNA genes or affect enzymes involved in miRNA processing, thereby perturbing miRNA maturation [43]. The present study shows that HD patients exhibited lower expression of miRNA-126-3p, -130a-3p, -146a-5p, -223-3p, -let7a-5p and -let7b-5p as compared with healthy controls. However, there were no significant changes observed during the whole follow-up period. Previous studies have indicated that these miRNAs are essential modulators of inflammatory and immune responses and contribute to CVD in ESRD [44, 45]. As evidenced by previous studies [46, 47], miRNA-126-3p is recognized as a marker of endothelial dysfunction and vascular inflammation. Similarly, deregulation of miRNA-223-3p is associated with vascular complications in patients with ESRD [48]. A decrease in levels of both miRNAs have been described in HD patients [49, 50], which is in agreement with our results that show lower levels observed in those with CVD [51]. This finding highlights the potential of these miRNAs as diagnostic and prognostic biomarkers in managing ESRD-related vascular disease. Surprisingly, to date, these miRNAs have not been regularly considered as biomarkers in this context.

In analysing the data by gender, we found no significant differences in the evaluated parameters between males and females, except for GDF-15 levels. Specifically, GDF-15 levels were significantly higher in males than in females, which aligns with findings from previous studies [52]. However, it is important to note that our study focuses on a population undergoing HD, unlike previous studies that involved subjects with normal kidney function, potentially offering new insights into GDF-15 in patients with impaired kidney function.

Despite the relevance of the results presented, which lead to reflection on the feasibility of including new biomarkers in CKD and its derived complications, the present study has some limitations that deserve to be mentioned. One limitation of this study is the inability to assess whether our biomarkers can predict prognosis in ESRD patients undergoing routine HD, due to the small sample size, which precludes such analysis. However, the methodological approach of using each patient as their own control helps to support the obtained findings. Another limitation is the geographic confinement of the study area, which predominantly includes Caucasian patients, making the generalization of our findings to other ethnicities unclear. Despite this, conducting the study within a clearly defined geographic area ensures that all the data come from a uniform well-structured database, thereby enhancing the reliability of the results.

In conclusion, this study provides valuable insights into the impact of the initiation of HD on new circulating inflammation and oxidative stress biomarkers. Our findings highlight significant changes in the inflammatory profile of patients that begin chronic HD, including an increase in the percentage of activated monocytes and the identification of new markers of inflammation and oxidative stress. These findings not only reflect the presence of subclinical inflammation in kidney disease but also suggest a very likely significant contribution to cardiovascular risk. Understanding these changes is essential for optimizing the management of ESRD patients and improving the overall efficacy of HD as a therapeutic intervention.

## Supplementary Material

sfaf223_Supplemental_Files

## Data Availability

All data generated or analysed during this study are included in this article. Further inquiries can be directed to the corresponding author.
