# Supplementary material for: New biomarkers of inflammation associated with haemodialysis
Source: Clin Kidney J. 2025 Jul 10;18(8):sfaf223. doi: 10.1093/ckj/sfaf223 (PMC12358798; doi:10.1093/ckj/sfaf223)
Supplement: sfaf223_Supplemental_Files [file sfaf223_supplemental_files.zip › 62 SUPPLEMENTARY DATA.docx]

**SUPPLEMENTARY DATA**

**Manuscript Title: New biomarkers of inflammation associated to hemodialysis.**

**Authors:** Fátima Guerrero,^1^*^#^ Andrés Carmona,^1^* Maria Jose Jiménez,^1^ Fran Ariza,^1,2^ Teresa Obrero,^1^ Isabel Berdud,^2^ Carolina Carrillo-Carrión,^3#^ Mariano Rodríguez,^1^ Sagrario Soriano,^1,4^ Juan R. Muñoz-Castañeda,^1,4†^ Alejandro Martín-Malo.^1†^

**Affilliation:**

1 Maimonides Biomedical Research Institute of Cordoba (IMIBIC), University of Córdoba, Reina Sofía University Hospital, Córdoba, Spain.

2 Dialysis satellite unit Fresenius Medical Care Services Andalucía, Córdoba, Spain

3 Institute for Chemical Research (IIQ), CSIC-University of Seville, Seville, Spain.

4 Nephrology Service, Reina Sofia University Hospital, Córdoba, Spain.

∗Fatima Guerrero and Andres Carmona share first authorship.

†Alejandro Martin-Malo and Juan R. Muñoz-Castañeda share last/senior authorship.

#**Correspondence:**

Fátima Guerrero

Avda/ Menendez Pidal s/n

+34 957 21 37 80

Email: [fatima.guerrero@imibic.org](mailto:fatima.guerrero@imibic.org)

Carolina Carrillo-Carrión

Avda/ Americo Vespucio 49

+ 34 954 48 95 56

Email: [carolina.carrillo@csic.es](mailto:carolina.carrillo@csic.es)

**Running head:** Inflammation biomarkers in hemodialysis.

**Figure S1. Monocyte subset.** A) CD14 high/SSC-A (side scatter area) was used to identify monocyte cells among other leucocytes. B) Plot of the SSC-A versus SSC-H and C) FSC-A versus FSC-H reveals that single cells align in one diagonal pattern, demonstrating the absence of doublets within this population. D and E) Forward and side scatter area (FSC-A/SSC-A) identified typical and atypical monocytes from single cells. F and H) Expression of surface markers CD14 and CD16 in typical monocytes. G and I) Expression of surface markers CD14 and CD16 in atypical monocytes.
